# Cardiac senescence is alleviated by the natural flavone acacetin via enhancing mitophagy

**DOI:** 10.18632/aging.203163

**Published:** 2021-06-27

**Authors:** Yi-Xiang Hong, Wei-Yin Wu, Fei Song, Chan Wu, Gui-Rong Li, Yan Wang

**Affiliations:** 1 Xiamen Cardiovascular Hospital, School of Medicine, Xiamen University, Xiamen, Fujian, China; 2 Nanjing Amazigh Pharma Ltd., Nanjing, Jiangsu, China

**Keywords:** cardiac senescence, telomere length shortening, mitophagy, Sirt1, Sirt6, pAMPK

## Abstract

Cardiac senescence is associated with cardiomyopathy which is a degenerative disease in the aging process of the elderly. The present study investigates using multiple experimental approaches whether the natural flavone acacetin could attenuate myocardial senescence in C57/BL6 mice and H9C2 rat cardiac cells induced by D-galactose. We found that the impaired heart function in D-galactose-induced accelerated aging mice was improved by oral acacetin treatment in a dose-dependent manner. Acacetin significantly countered the increased serum advanced glycation end products, the myocardial telomere length shortening, the increased cellular senescence marker proteins p21 and p53, and the reduced mitophagy signaling proteins PINK1/Parkin and Sirt6 expression in aging mice. In H9C2 rat cardiac cells, acacetin alleviated cell senescence induced by D-galactose in a concentration-dependent manner. Acacetin decreased p21 and p53 expression, up-regulated PINK1/Parkin, LC3II/LC3I ratio, pLKB1, pAMPK and Sirt6, and reversed the depolarized mitochondrial membrane potential in aging cardiac cells. Mitophagy inhibition with 3-methyladenine or silencing Sirt6 abolished the protective effects of acacetin against cardiac senescence. Further analysis revealed that acacetin effect on Sirt6 was mediated by Sirt1 activation and increase of NAD^+^/NADH ratio. These results demonstrate that acacetin significantly inhibits *in vivo* and *in vitro* cardiac senescence induced by D-galactose via Sirt1-mediated activation of Sirt6/AMPK signaling pathway, thereby enhancing mitophagy and preserving mitochondrial function, which suggests that acacetin may be a drug candidate for treating cardiovascular disorders related to aging.

## INTRODUCTION

Cellular senescence is associated with increased tissue remodeling during development and after injury, decreased regenerative potential and promotion of inflammation and tumorigenesis in aged organisms [1]. The aged organisms are characterized by accumulation of senescent cells and by progressive loss of physiological integrity with impaired function and increased vulnerability to death. The molecular biological hallmarks of aging include unstable genome, telomere shortening, changes in epigenetic, proteostasis and nutrient sensing, mitochondrial dysfunction, stem cell exhaustion, and altered intercellular communication [2]. Aging is believed to be an independent risk factor of cardiovascular diseases and cardiac aging is related to deficiencies in myocardial mitochondrial metabolism and mitochondrial respiration [3, 4]. Mitochondrial dysfunction and impairment of mitochondrial-autophagy (i.e. mitophagy) have been considered to be important components of the cellular processes of myocardial aging [5–7].

In hearts, the mitochondrial kinase PINK1 (PTEN-induced kinase 1) accumulates on the outer mitochondrial membrane and initiates Parkin (E3 ubiquitin ligase) translocation [8, 9] to regulate mitochondrial quality control and promote mitophagy. When mitochondria are impaired and lose membrane potential, PINK1 recruits Parkin to target these mitochondria for autophagic removal [10]. Increasing evidence shows that Parkin deficiency accelerates aging phenotype and cause accumulation of aberrant mitochondria in aging heart [11–13], while cardiac specific overexpression of Parkin can ameliorate cardiac aging by enhancing mitochondrial turnover [13]. Therefore, PINK1/Parkin play an important role in the induction of cardiac mitophagy [9, 14].

Anti-aging strategies can improve both health and lifespan. Experimental studies reported that the enhancement of autophagy can increase the lifespan of various organisms ranging from worms to mice [15, 16]. Our recent studies have demonstrated the natural flavone acacetin provides cardioprotection against ischemia/reperfusion injury [17], hypoxia/reoxygenation injury [18], and doxorubicin cardiotoxicity [19]. The present study investigates whether acacetin could inhibit cardiac senescence in D-galactose-induced *in vivo* and *in vitro* accelerated aging models [20, 21] with multiple experimental approaches including echocardiography, biochemical and molecular biological technologies.

## RESULTS

### Acacetin improves heart function of D-galactose-induced accelerated aging mice

**Figure 1:**
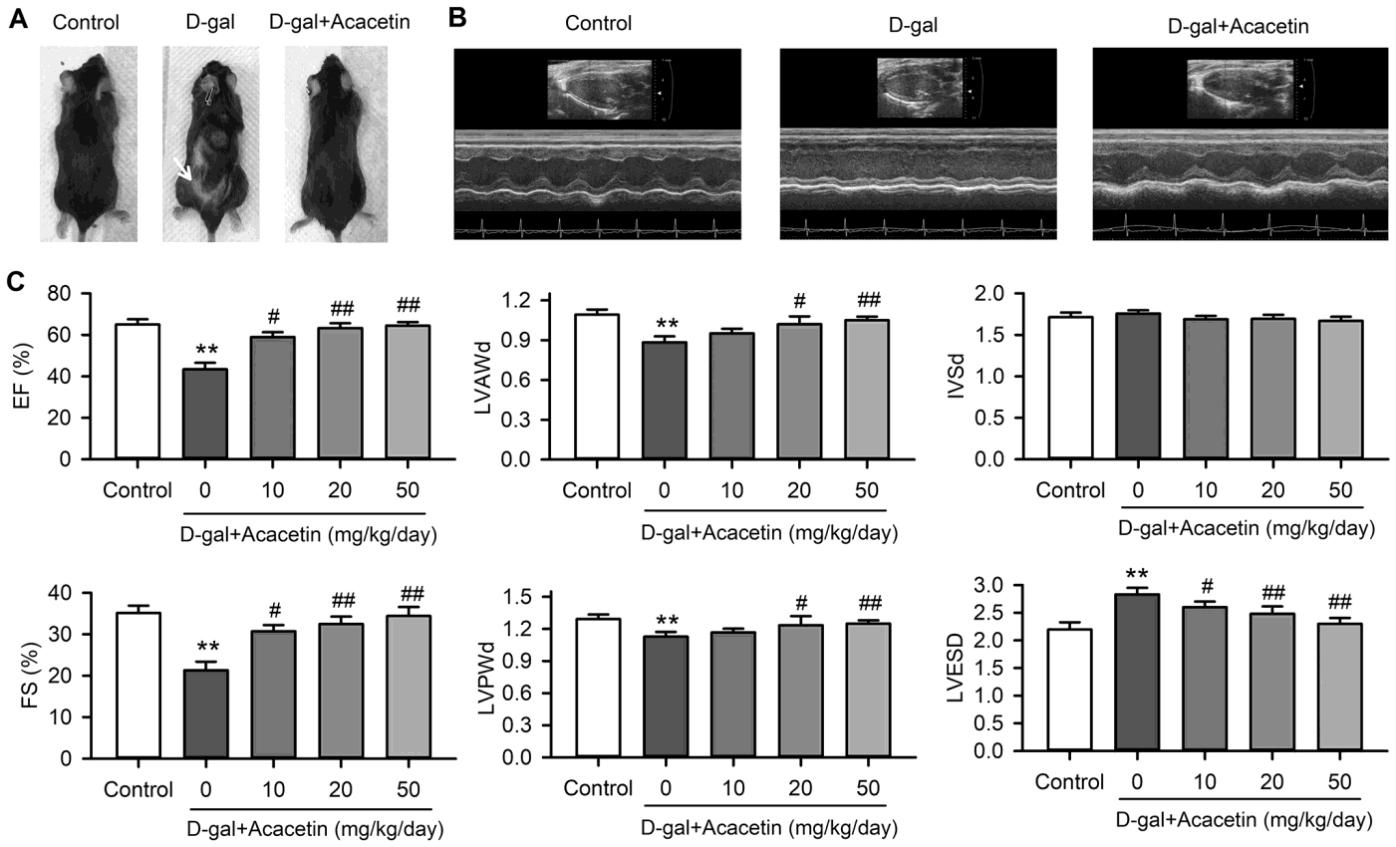
**Effects of acacetin on cardiac function in a mouse aging model induced by D-galactose.** (**A**). Back hair loss (white arrow) was observed in mice with subcutaneous injection of D-galactose (D-gal, 150 mg/kg/day), but not in D-galactose mice with oral acacetin (50 mg/kg/day). (**B**) Representative echocardiography images of control mouse, D-galactose mouse and D-galactose mouse with oral acacetin (50 mg/kg/day). (**C**) Echocardiograph parameters: left ventricular ejection fraction (EF), fractional shortening (FS), diastolic left ventricular anterior wall thickness (LVAWd), diastolic left ventricular posterior wall thickness (LVPWd), thickness of interventricular septal end diastole (IVSd), and left ventricular end-systolic diameter (LVESD) in mice without (control) or with D-galactose or D-galactose plus oral acacetin 10, 20 or 50 mg/kg/day (*n* = 10-12, ^**^P < 0.01 vs. control group. ^#^*P* < 0.05, ^##^*P* < 0.01 vs D-gal group).

Subcutaneous injection of D-galactose (150 mg/kg) resulted in back hair loss was in 50% of aging C57/BL6 mice (6 of 12 mice), but not in mice with oral acacetin administration at 10, 20 or 50 mg/kg/day (Figure 1A, Supplementary Figure 1). Serum advanced glycation end products (AGEs) was increased in D-galactose-induced accelerated aging mice to 0.91 ± 0.17 μg/mL from 0.52 ± 0.13 μg/mL in control (*n* = 8, *P* < 0.05), which was decreased to 0.68 ± 0.12 μg /mL, 0.61 ± 0.11 μg/mL, and 0.51 ± 0.18 μg/mL in mice treated with 10, 20 or 50 mg/kg/day of acacetin, respectively (*n* = 7. *P* < 0.05 vs. D-galactose alone). These results suggest that acacetin may prevent D-galactose-induced hair loss and reduce the formation of AGEs.

Heart function of the experimental animals was evaluated using echocardiography (Figure 1B). The heart ejection fraction (EF) and the fractional shortening (FS) were decreased from 65.1 ± 2.5% and 35.1 ± 1.8% in control mice (*n* = 12) to 43.4 ± 3.1% and 21.3 ± 2.1% (*n* = 10, *P* < 0.01 vs. control) in aging mice induced by D-galactose with decreased thickness of the left ventricular anterior and posterior wall and increased systolic left ventricular inner diameter (Figure 1C). Intriguingly, the reduced EF and FS were countered in mice treated with oral acacetin administration in a dose-dependent manner, and the decreased thickness of diastolic left ventricular anterior wall thickness (LVAWd) and diastolic left ventricular posterior wall thickness (LVPWd) as well as the increased left ventricular end-systolic inner diameter (LVESD) were reversed by acacetin administration. In addition, heart weight (HW)/body weight (BW) ratio was significantly decreased with reduced thickness of left ventricular wall in D-galactose aging mice, which were improved in the aging mice treated with acacetin in a dose-dependent manner (Supplementary Figure 2). These results suggest that the reduction of heart function is likely resulted from a dilated cardiomyopathy in aging mice induced by D-galactose, and acacetin improves the heart function in a dose-dependent manner.

### Acacetin decreases myocardial senescence markers and increases autophagy proteins in aging mouse model induced by D-galactose

**Figure 2:**
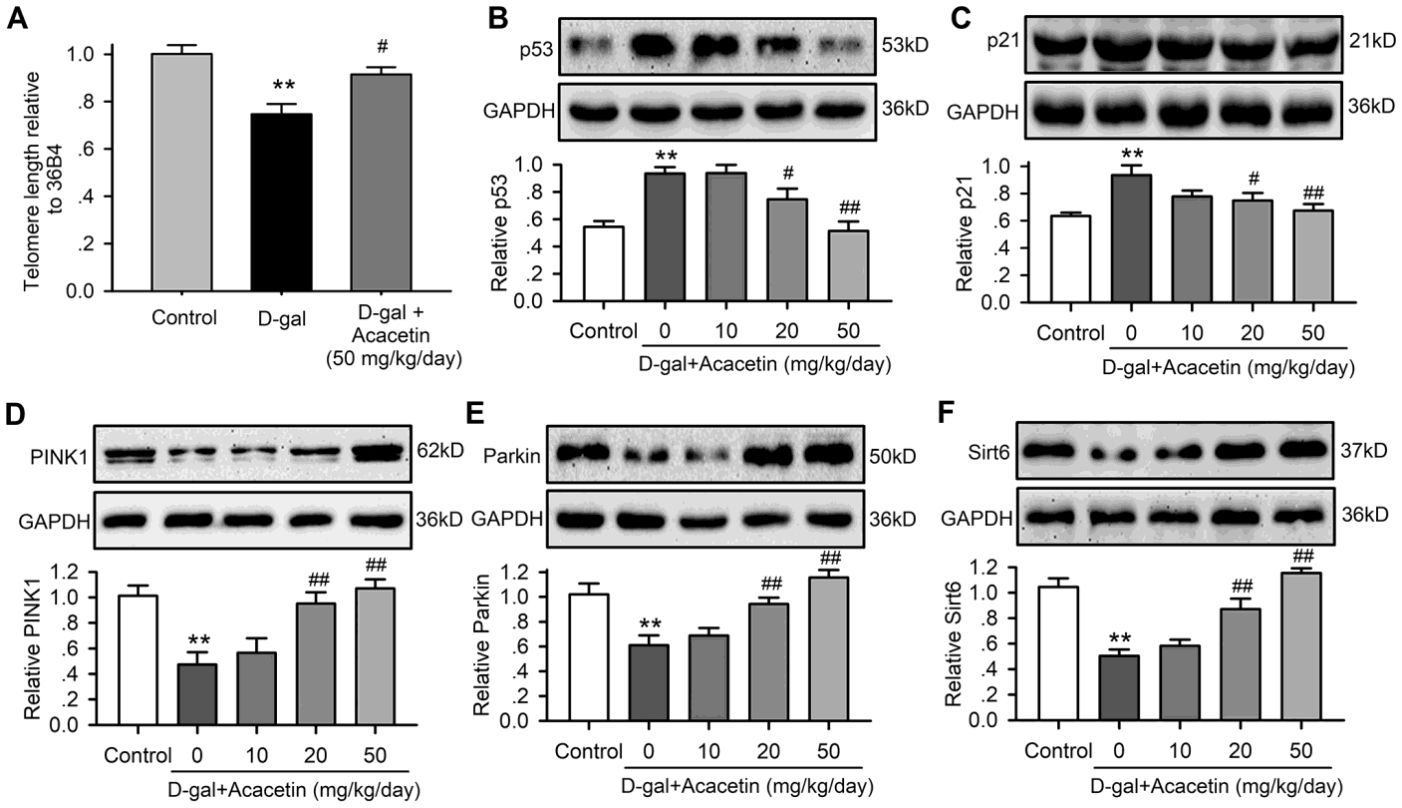
**Acacetin effects on myocardial telomere length and proteins related to aging and autophagy in senescence mouse model induced by D-galactose.** (**A**) Myocardial telomere length determined by qRT-PCR in mice treated without (control) or with D-galactose (D-gal, 150 mg/kg/day) or D-galactose with acacetin (50 mg/kg/day). (**B**) Western blots and relative levels of p53 protein in ventricular tissues of mice treated without (control) or with D-galactose or D-galactose with oral 10, 20 or 50 mg/kg acacetin. (**C**) Western blots and relative levels of p21 protein in ventricular tissues of mice treated as in (**B**). (**D**) Western blots and relative levels of PINK1 protein in ventricular tissues of mice treated as in (**B**). (**E**) Western blots and relative levels of Parkin protein in ventricular tissues of mice treated as in (**B**). (**F**) Western blots and relative levels of Sirt6 protein in ventricular tissues of mice treated as in (**B**) (*n* = 6, ^**^P < 0.01 vs. control. ^#^*P* < 0.05, ^##^*P* < 0.01 vs D-gal).

Cardiac senescence is characterized by telomere length shortening [22, 23] and an increase in aging markers p53 and p21 with decreased autophagy in aging animals [20, 24]. We therefore determined the telomere length with qPCR (Supplementary Figure 3) using procedures described previously [25], and the protein levels of the senescence markers p53 and p21 with western blot analysis in myocardial tissues of C57/BL6 mice treated with D-galactose or D-galactose plus acacetin (Figure 2) to determine whether acacetin-induced improvement of the impaired heart function was related to inhibiting cardiac senescence. It is interesting to note that telomere length was shortened (Figure 2A), and p53 (Figure 2B) and p21 (Figure 2C) were significantly increased in mice treated with D-galactose, these effects were reversed in D-galactose mice treated with acacetin. In addition, the mitophagy core machinery kinases PINK1 and Parkin were downregulated in mice treated with D-galactose, and the decreased PINK1 and Parkin were upregulated in D-galactose mice treated with acacetin in a dose-dependent manner (Figure 2D, 2E). Moreover, myocardial Sirt6 expression was reduced in mice with D-galactose, which was also recovered in mice treated with acacetin. These results suggest that cardiac senescence induced by D-galactose can be countered by acacetin.

### Effects of acacetin on cardiac senescence induced by D-galactose

**Figure 3:**
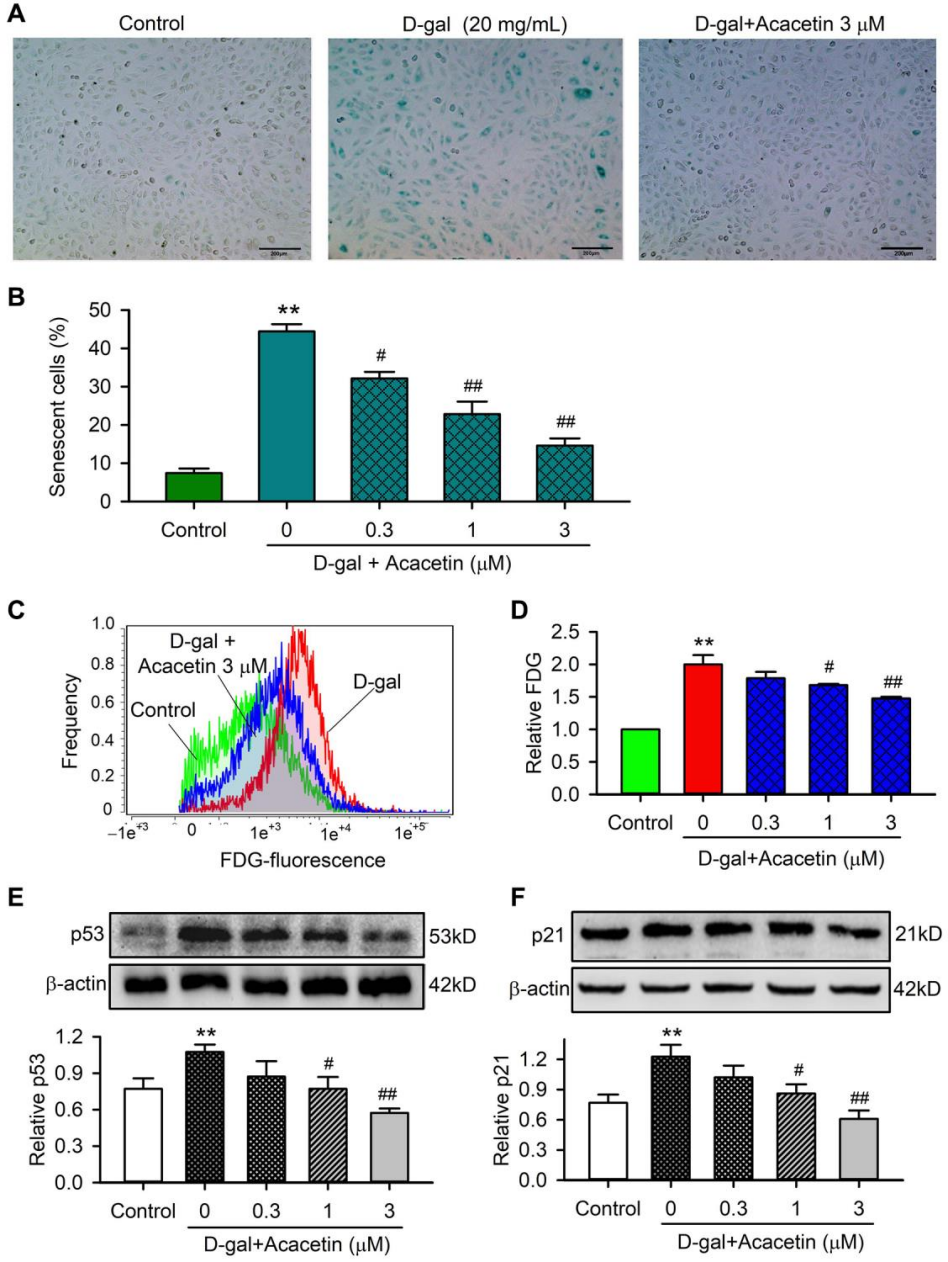
**Effects of acacetin on cellular senescence induced by D-galactose in H9C2 cardiac cells.** (**A**) Representative images of senescence-associated-β-galactosidase (SA-β-gal) staining for senescent cells in H9C2 cardiac cells treated without (control) or with D-galactose (D-gal, 20 mg/mL) in the absence and presence of 3 μM acacetin for 72 h. (**B**) Percentage of SA-β-gal-positive senescent cell number in cardiac cells treated without (control) or with D-gal (20 mg/mL) in the absence and presence of 0.3, 1 or 3 μM acacetin for 72 h. (**C**) Flow cytometry graphs for determining activity of fluorescein di-β-D-galactopyranoside (FDG) in cells treated as in (**A**). (**D**) Relative FDG level in cardiac cells treated as in (**B**). (**E**) Western blot and relative level of p53 protein in cells treated as in (**B**). (**F**) Western blot and relative level of p21 in cells treated as in (**B**). (*n* = 5, ^**^P < 0.01 vs. control. ^#^*P* < 0.05, ^##^*P* < 0.01 vs. D-gal).

To investigate the potential molecular mechanism involved in acacetin-mitigation on reduced cardiac function observed in D-galactose-induced aging mice, H9C2 cardiac cells were exposed to D-galactose (20 mg/mL) for 72 h in the absence or presence of acacetin (0.3, 1 or 3 μM). Figure 3 illustrates the effects of acacetin on D-galactose-induced cardiac senescence determined by senescence-associated-β-galactosidase (SA-β-gal) staining and flow cytometry with fluorescein di-β-D-galactopyranoside staining and western blot analysis for protein expression of the aging markers p53 and p21. The percentage of SA-β-gal positive (senescence) cells was increased by D-galactose from 7.4 ± 1.2% of control to 44.4 ± 1.9% which was reduced by acacetin treatment in a concentration-dependent manner (to 14.6 ± 1.8% at 3 μM acacetin) (Figure 3C). Flow cytometry analysis revealed that activity of β-galactosidase was greatly increased by D-galactose and decreased by acacetin (Figure 3C, 3D). The aging marker proteins p53 and p21 were upregulated by D-galactose, these upregulations were inhibited by acacetin treatment in a concentration-dependent manner (Figure 3E, 3F). These results demonstrate that D-galactose-induced senescence in H9C2 cardiac cells is mitigated by acacetin.

### Acacetin reverses the decrease in cardiac mitophagy by D-galactose

**Figure 4:**
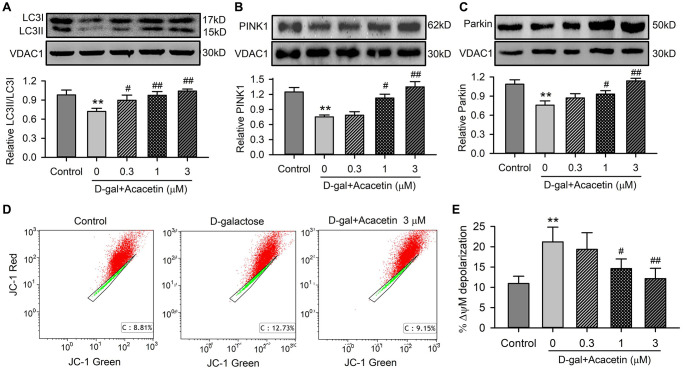
**Effects of acacetin on mitophagy kinase proteins and mitochondrial membrane potential in H9C2 cardiac cells.** (**A**) Western blot of mitochondrial LC3II, LC3I and relative LC3II/LC3I ratio in mitochondrial proteins of cells treated without (control) or with D-galactose (D-gal) exposure or D-galactose plus 0.3, 1 or 3 μM acacetin for 72 h. (**B**) Western blot and relative level of mitochondrial PINK1 in cells treated as in (**A**). (**C**) Western blot and relative level of mitochondrial Parkin in cells treated as in (**A**). (**D**) Representative graphs of flow cytometry for determining mitochondrial membrane potential in cells stained with JC-1 (2 μM) in cells treated without (control) or with D-galactose (20 mg/mL) or D-galactose plus 3 μM acacetin. (**E**) Percentage of mitochondrial membrane potential depolarization (δψM) in cells treated as A. (*n* = 5, ^**^P< 0.01 vs. control. ^#^*P* < 0.05, ^##^*P* < 0.01 vs. D-gal).

Cell autophagy and mitophagy play a vital role in regulating cardiac senescence [26], we therefore determined the effects of acacetin on the cytosolic autophagosome marker kinases LC3I and LC3II, and the mitophagy kinases PINK1 and Parkin in mitochondrial proteins of H9C2 cardiac cells treated with D-galactose. D-galactose remarkably decreased the ratio of LC3II/LC3I (Figure 4A), and the mitophagy marker kinases PINK1 (Figure 4B) and Parkin (Figure 4C). Acacetin upregulated LC3II/LC3I ratio, PINK1 and Parkin in a concentration-dependent manner. Flow cytometry analysis revealed that aging cardiac cells induced by D-galactose showed a depolarized mitochondrial membrane potential (Figure 4D), which was reversed by acacetin in a concentration-dependent manner (Figure 4E). These results suggest that acacetin-induced improvement of cardiac senescence may be related to maintenance of integral mitochondrial function by enhancing mitophagy.

**Figure 5:**
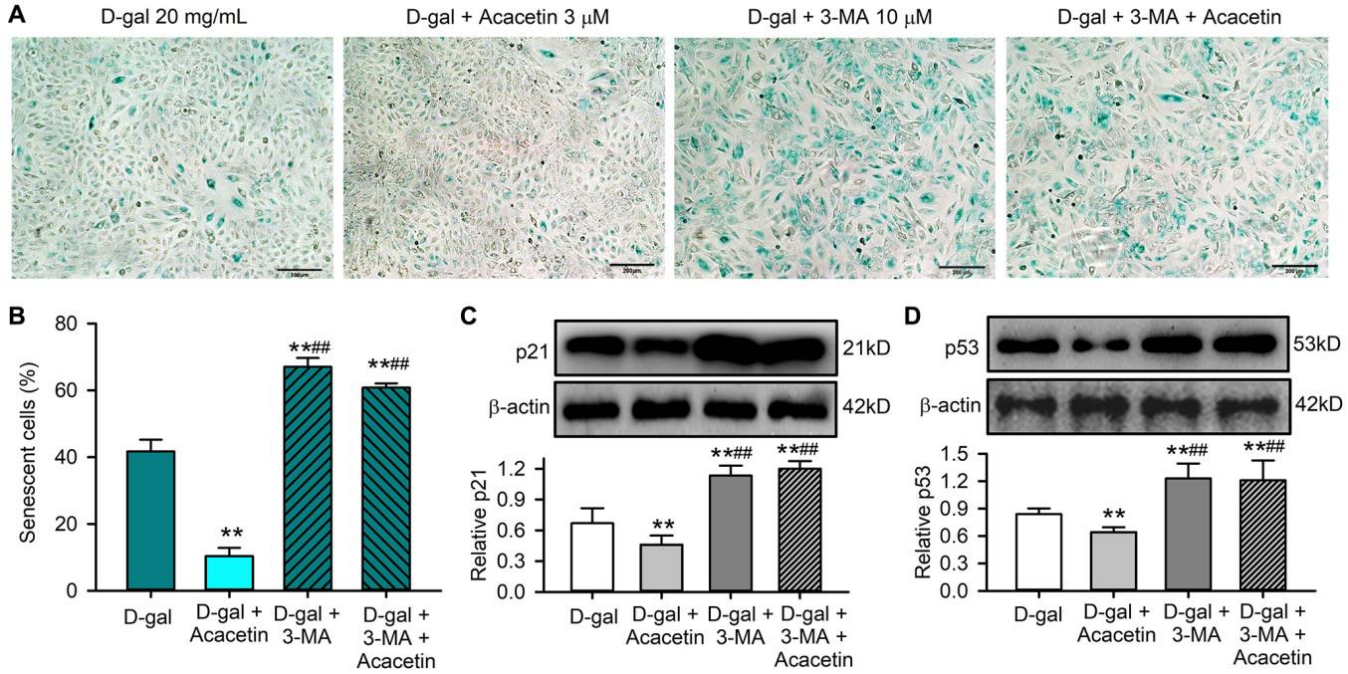
**The autophagy inhibitor 3-methyladenine abolished the protective effect of acacetin against H9C2 cell senescence induced by D-galactose.** (**A**) Representative images of SA-β-gal staining for senescent cells in H9C2 cardiac cells treated with D-galactose (D-gal) in the absence or presence of 3 μM acacetin or 10 μM 3-methyladenine (3-MA) with or without 3 μM acacetin for 72 h. (**B**) Percentage of senescent cells in cells treated as in (**A**). (**C**) Western blots and relative level of p21 protein in cells treated as in (**A**). (**D**) Western blots and relative level of p53 protein in cells treated as in (**A**). (*n* = 4, ^**^P < 0.01 vs. control. ^##^*P* < 0.01 vs. D-gal).

If the improvement of cardiac senescence induced by acacetin is due to enhancing mitophagy, the effect would be decreased by the autophagy inhibitor 3-methyladenine. Figure 5 illustrates the effects of acacetin on cardiac senescence induced by D-galactose in the absence and presence of 10 μM 3-methyladenine in H9C2 cardiac cells. Senescent cells were stained by SA-β-gal. 3-Methyladenine exacerbated cellular senescence, and abolished the acacetin-induced decrease of cardiac cell senescence (Figure 5A, 5B) and senescence marker proteins (Figure 5C, 5D). These results indicate that the anti-senescence effect of acacetin is related to promoting cardiac mitophagy.

### Acacetin protects against cardiac senescence induced by activating Sirt6

**Figure 6:**
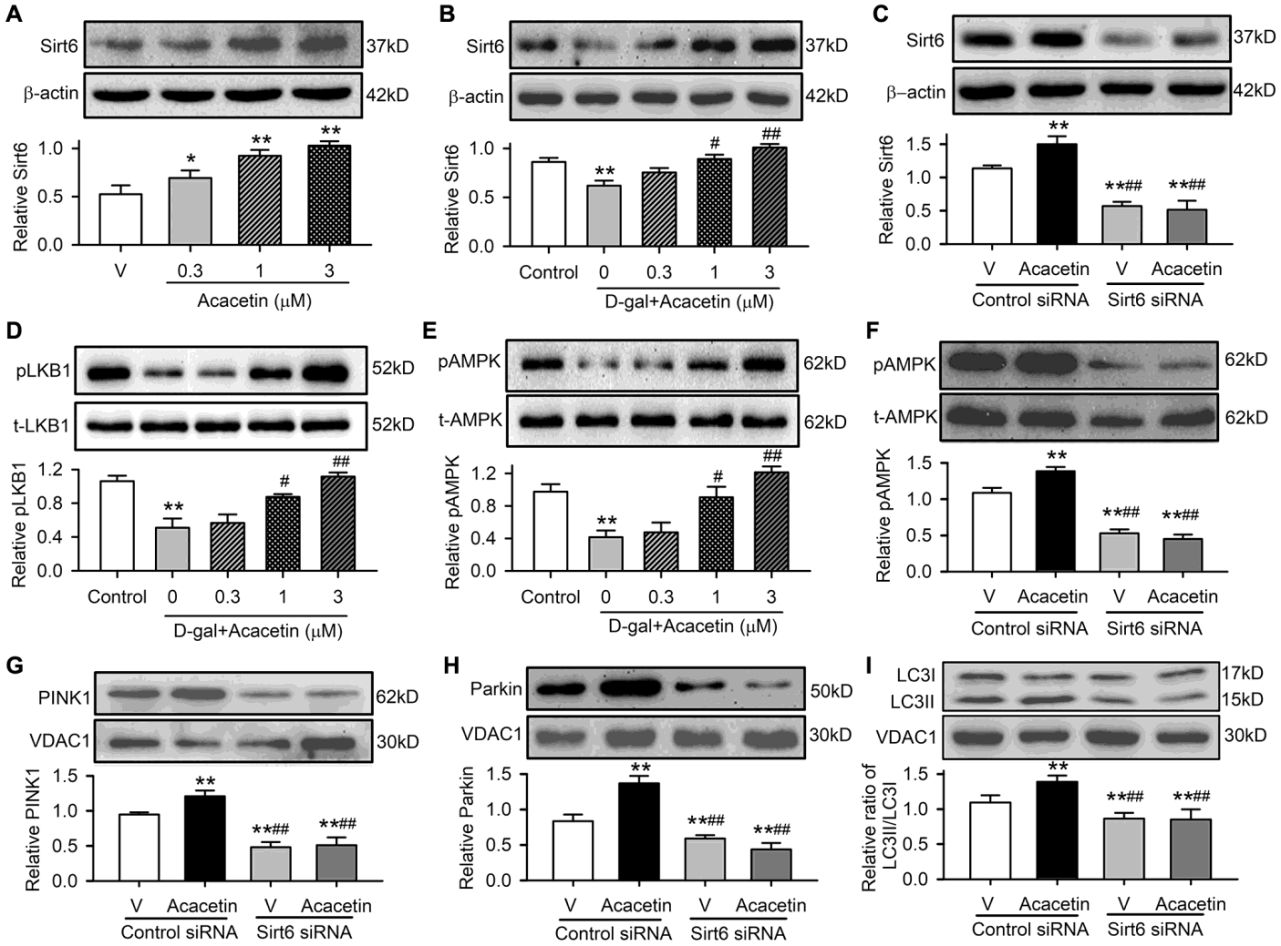
**Sirt6 mediates acacetin-induced reversal of the downregulation of pLKB1, pAMPK and mitophagy signaling in H9C2 cells by D-galactose.** (**A**) Western blots and relative levels of Sirt6 protein in cells treated without (V, vehicle) or with 0.3, 1 or 3 μM) for 72 h. (**B**) Western blots and relative levels of Sirt6 in cells treated without (control) or with D-galactose (D-gal, 20 mg/mL) or D-galactose plus acacetin (0.3, 1 or 3 μM) for 72 h. (**C**) Western blots and relative levels of Sirt6 protein in cells transfected with control siRNA or Sirt6 siRNA in the absence (V, vehicle) or presence of 3 μM acacetin. (**D**) Western blots and relative levels of pLKB1 in cells treated as in (**B**). (**E**) Western blots and relative levels of pAMPK in cells treated as in (**B**). (**F**) Western blots and relative levels of pAMPK in cells treated as in (**C**). (**G**) Western blots and relative levels of PINK1 in cells treated as in (**C**). (**H**) Western blots and relative levels of Parkin in cells treated as in (**C**). (**I**) Western blots and relative levels of LC3II/LC3I ratio in cells treated as in (**C**). (*n* = 5, ^*^P < 0.05, ^**^P < 0.01 vs. vehicle, control or vehicle of control siRNA; ^#^*P* < 0.05, ^##^*P* < 0.01 vs. D-gal or control siRNA with acacetin).

Sirt6 plays an important role regulating aging and aging-related disorders [27]. This is supported by the observation in the present study, in which myocardial Sirt6 was downregulated in mice with D-galactose and was upregulated by acacetin treatment (Figure 2F). We therefore determined how Sirt6 is involved in acacetin protection against cardiac senescence in H9C2 cardiac cells (Figure 6). Acacetin enhanced Sirt6 expression in H9C2 cardiac cells (Figure 6A), and reversed D-galactose-induced Sirt6 reduction (Figure 6B) in a concentration-dependent manner. However, this was not observed in cells transfected with Sirt6 siRNA (Figure 6C). Acacetin also reversed D-galactose-induced reduction of pLKB1 (Figure 6D) and pAMPK (Figure 6E), and the effect was abolished in cells with silenced Sirt6 (Figure 6F). Moreover, silencing Sirt6 not only decreased mitophagy-associated proteins PINK, Parkin and LC3II/LC3I ratio, but also abolished acacetin-induced enhancement of these proteins (Figure 6G–6I). These results indicate that Sirt6 plays a crucial role in mediating the protection of acacetin against cardiac senescence by enhancing mitophagy.

**Figure 7:**
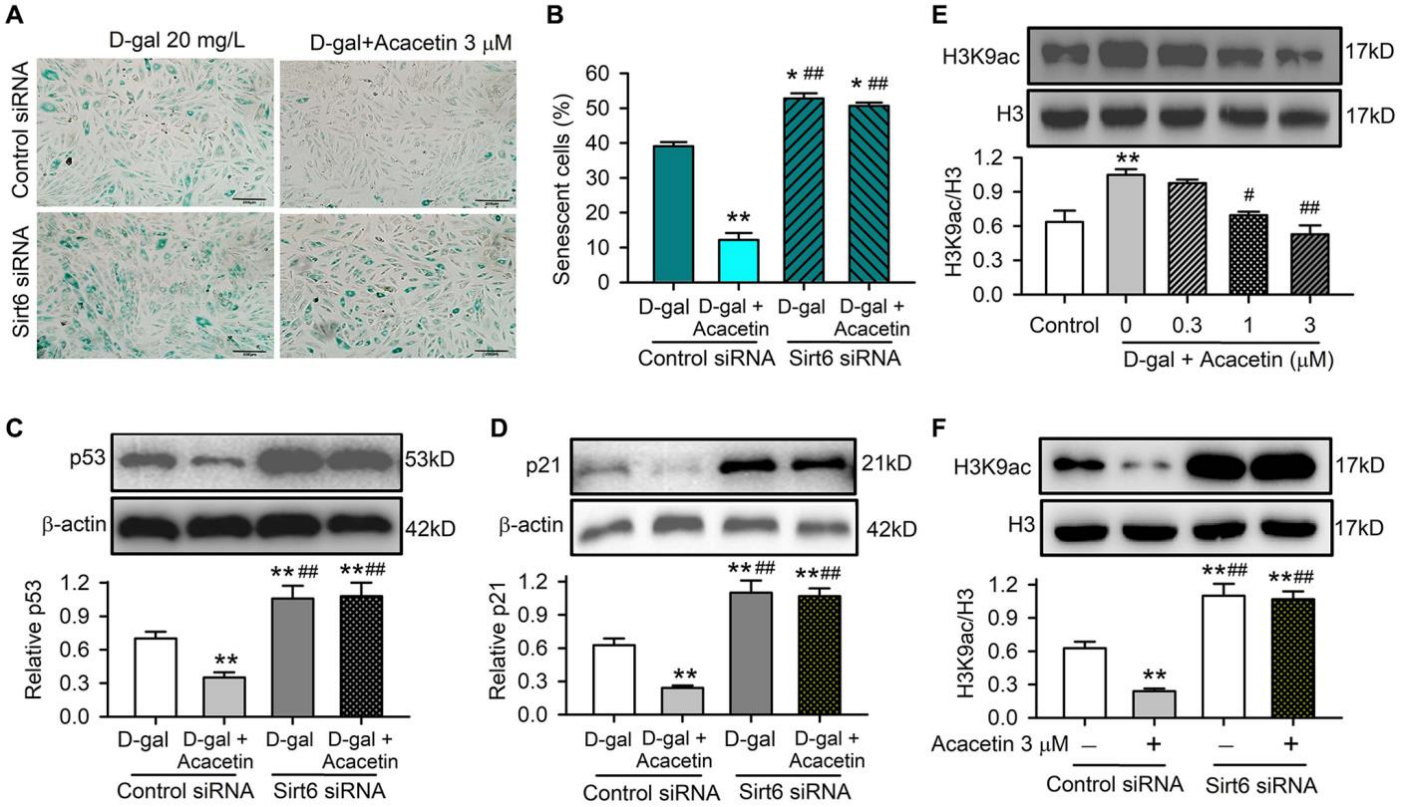
**Acacetin-induced activation of Sirt6 mediates the protection against cardiac senescence by inhibiting protein acetylation.** (**A**) Representative images of SA-β-gal staining for senescent cells in H9C2 cardiac cells transfected with control siRNA or Sirt6 siRNA in the absence or presence of 3 μM acacetin for 72 h. (**B**) Percentage of senescent cell number in cells treated as in (**A**). (**C**) Western blots and relative levels of p53 in cells treated as in (**A**). (**D**) Western blots and relative levels of p21 in cells treated as in (**A**) (*n* = 4-6, ^**^P < 0.01 vs. D-gal alone, ^##^*P* < 0.01 vs. control siRNA with 3 μM acacetin). (**E**) Western blots and relative levels of H3K9ac in cells treated without (control) or with D-galactose (D-gal, 20 mg/mL) or D-galactose plus acacetin (0.3, 1 or 3 μM) for 72 h (*n* = 4, ^**^P < 0.01 vs. control, ^#^*P* < 0.05, ^##^*P* < 0.01 vs. with D-galactose alone). (**F**) Western blots and relative levels of H3K9ac in cells treated as in (**A**) (*n* = 4–6, ^**^P < 0.01 vs. D-gal alone, ^##^*P* < 0.01 vs. control siRNA with 3 μM acacetin).

Furthermore, cardiac senescence induced by D-galactose could be reduced by acacetin in H9C2 cardiac cells transfected with control siRNA, but not in cells transfected with Sirt6 siRNA (Figure 7A, 7B). The cardiac senescence markers p53 and p21 were reduced by acacetin in cells transfected with control siRNA, but not in cells with silenced Sirt6 (Figure 7C, 7D). These results indicate that Sirt6 plays a crucial role in mediating the protection of acacetin against cardiac senescence by enhancing mitophagy.

Sirt6 is a histone deacetylase that plays an essential role in regulating mitochondrial function in different cell types by deacetylation. The acetylated histone H3 lysine 9 (H3K9ac), which is correlated with transcriptional activation and chromatin reassembly during DNA replication, is a typical physiological substrate of Sirt6 [28]. We therefore determined the expression of H3K9ac in H9C2 cardiac cells with D-galactose- induced senescence with or without acacetin administration. H3K9ac was significantly increased in D-galactose-induced senescent cells, and acacetin (1–3 μM) reduced the increase of H3K9ac in a concentration dependent manner (Figure 7E). In another set of experiment with normal cell culture, H3K9ac was reduced by 3 μM acacetin in cells transfected with control siRNA; while H3K9ac was remarkably increased, and acacetin-induced reduction was abolished in cells transfected with Sirt6 siRNA (Figure 7F). These results suggest that the anti-senescence effect of acacetin correlates to the inhibition of histone acetylation through Sirt6 activation.

Our recent studies have shown that acacetin protects cardiac cells against doxorubicin-induced injury and endothelial cells against high glucose injury by activating Sirt1 followed by increasing pAMPK and/or Sirt3 [19, 29]. To determine whether beneficial effects of acacetin are mediated by other sirtuins, we determined the effects of acacetin on Sirt2, Sirt5, Sirt7, and also Sirt1 expression in H9C2 cardiac cells. Acacetin significantly increased Sirt1 expression, but did not increase Sirt2, Sirt5, or Sirt7 expression (Supplementary Figure 4).

**Figure 8:**
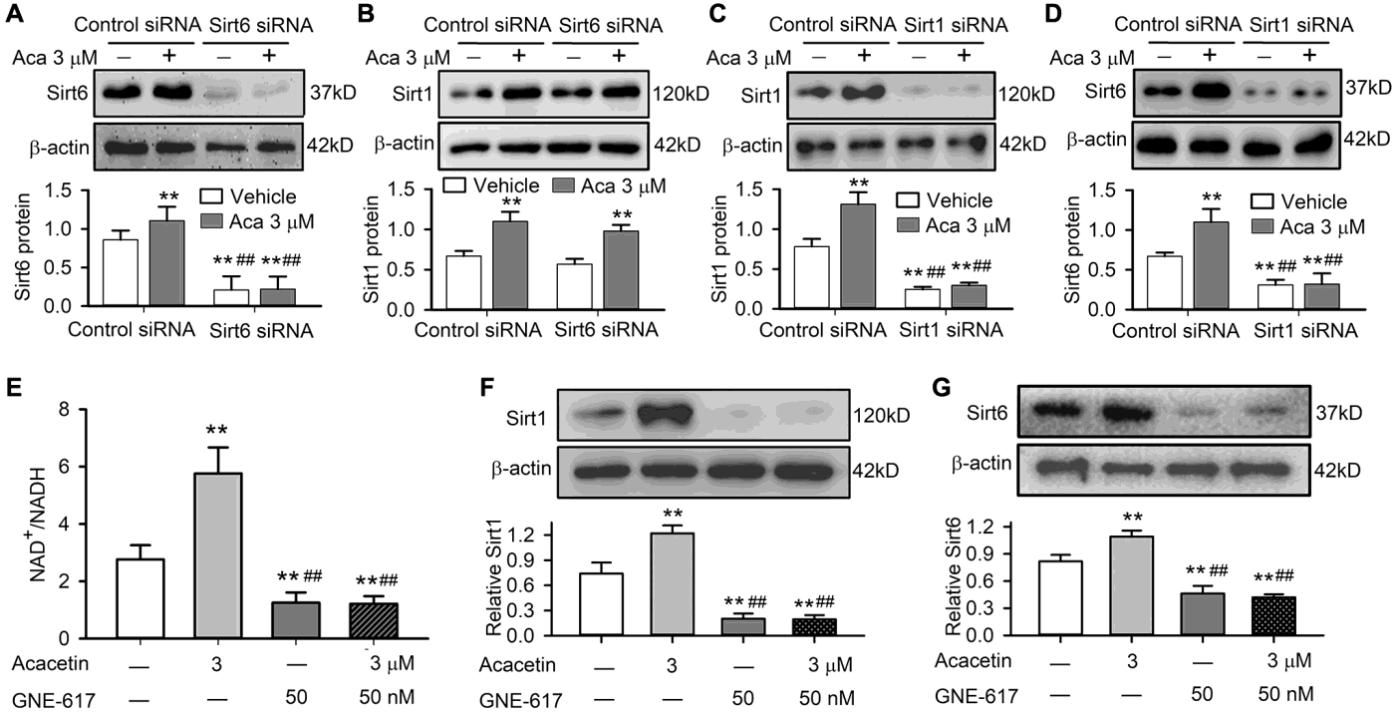
**Sirt1 mediates acacetin-induced Sirt6 activation by enhancing NAMPT and NAD^+^/NADH in H9C2 cardiac cells.** (**A**) Western blots and relative levels of Sirt6 in cells transfected with control siRNA or Sirt6 siRNA in the absence or presence of 3 μM acacetin (Aca) for 72 h. (**B**) Western blots and relative levels of Sirt1 in cells as treated in A. (**C**) Western blots and relative levels of Sirt1 in cells transfected with control siRNA or Sirt1 siRNA in the absence or presence of 3 μM acacetin for 72 h. (**D**) Western blots and relative levels of Sirt6 in cells as treated in C (*n* = 4–6, ^**^P < 0.01 vs. control siRNA, ^##^*P* < 0.01 vs. control siRNA with acacetin). (**E**) Acacetin-induced increase of NAD^+^/NADH ratio was prevented by NAMPT inhibitor GNE-617 (50 nM). (**F**) Acacetin-induced increase of Sirt1 protein was abolished by 50 nM GNE-617. (**G**) Acacetin-induced increase of Sirt6 protein was abolished by 50 nM GNE-617. (*n* = 4–6, ^**^P < 0.01 vs. control, ^##^*P* < 0.01 vs. acacetin alone).

Sirt1 is also involved in acacetin protection against D-galactose-induced senescence, and Sirt1 was downregulated in cardiac tissue of mice with D-galactose and acacetin administration reversed the reduced Sirt1 expression by acacetin in a dose-dependent manner (Supplementary Figure 5). The relation of Sirt6 to Sirt1 was determined in H9C2 cardiac cells transfected with Sirt6 siRNA or Sirt1 siRNA (Figure 8A–8D). It is interesting to note that silencing Sirt6 only reduced Sirt6 expression and abolished acacetin-induced increase of Sirt6, but had no effects on Sirt1 expression or acacetin-induced increase of Sirt1 (Figure 8A, 8B). However, silencing Sirt1 reduced both Sirt1 and Sirt6 expression and abolished acacetin-induced increase of Sirt1 and Sirt6 (Figure 8C, 8D). These results indicate that acacetin-induced Sirt6 increase is mediated by Sirt1 activation.

It is generally believed that mammalian sirtuins are regulated by the ratio of NAD^+^/NADH as well as activity of nicotinamide phosphoribosyltransferase (NAMPT) [30, 31]. We observed that NAMPT was reduced in cardiac tissue of mice with D-galactose and the reduction was reversed in mice with acacetin administration in a dose-dependent manner (Supplementary Figure 6). In addition, acacetin-induced Sirt1 and Sirt6 activations were found to be related to increase in NAD^+^/NADH ratio in H9C2 cardiac cells. Acacetin (3 μM) significantly increased NAD^+^/NADH ratio, while the NAMPT inhibitor GNE-617 (50 nM) [32] not only decreased NAD^+^/NADH ratio, but also abolished the effect of acacetin (Figure 8E). In addition, GNE-617 decreased both Sirt1 and Sirt6 protein level, and fully abolished acacetin-induced increase of Sirt1 and Sirt6 expressions (Figure 8F, 8G). These results indicate that activation of Sirt1 and Sirt6 is related to increasing NAD^+^/NADH ratio in H9C2 cardiac cells.

## DISCUSSION

It is well recognized that cardiovascular disease is a major cause of death worldwide. Aging is a strong risk factor for heart disease in the elderly population in which two-thirds of deaths are from cardiovascular disorders [33]. Demand for anti-aging strategies and treatments to improve health and lifespan is increasing [31, 34]. The present study demonstrates that the natural flavone acacetin attenuates cardiac senescence in D-galactose-induced accelerated aging mice in a dose-dependent manner and is associated with decreasing the serum AGEs, mitigating the telomere length shortening and reducing the increased cellular senescence marker proteins p21 and p53. Acacetin protection against cardiac senescence is correlated with enhancing the mitophagy proteins PINK1 and Park, and LC3II via Sirt1-mediated activation of Sirt6 and pAMPK. The results from this study suggest that acacetin may be a drug candidate for improving health and lifespan in the elderly population.

Acacetin is a natural flavone widely present in many plants, e.g. *Saussureae Involucratae Herba* (Snow Lotus) [35], *Carthamus tinctorius L*. [36], *Chrozophora tinctoria L*. [37], *Calea urticifolia* [38], etc. [39], which can also be synthesized in laboratories [40, 41]. Acacetin has diverse pharmacological activities, e.g. attenuating mice lipid accumulation [42] and endotoxin-induced acute lung injury [43], neuronal protection against ischemia/reperfusion injury [44], anti-cancer effects [45, 46], etc. We have previously demonstrated that acacetin has atrial-selective anti-atrial fibrillation effect [40, 41] by preferentially blocking atrial potassium channels including I_Kur_ (ultra-rapidly delayed rectifier potassium current) [40, 47], I_KACh_ (acetylcholine-activated potassium current), sKCa current (calcium-activated small-conductance potassium current) [48], and also I_to_ (transient-outward potassium current) [49]. In addition, acacetin provides strong protection against myocardial ischemia/reperfusion (or hypoxia/reoxygenation) injury by inhibiting inflammation, oxidation, and apoptosis via activating AMPK/Nrf2 signaling [17, 18], and reducing cardiotoxicity induced by the chemical therapy drug doxorubicin via activating Sirt1/AMPK signal molecules [19]. It has also been reported to protect against cardiac ischemic remodeling by activating MAPK and PI3K/Akt signal pathway [50]. This study demonstrates the new pharmacological effect that acacetin protects against D-galactose-induced cardiac senescence by enhancing mitophagy via Sirt1-mediated activation of Sir6/AMPK signals.

D-galactose is a reducing sugar present in the body that can be converted into aldose and hydroperoxide by galactose oxidase at high concentrations, resulting in accumulation of ROS. It can form galactitol by galactose reductase, which results in osmotic stress. It also reacts with the amino groups in proteins and peptides to form AGEs, which are significantly increased with further ROS communication and degenerative changes during aging [20, 51, 52]. Therefore, D-galactose is widely employed to artificially induce senescence *in vitro* and *in vivo* for anti-aging therapeutic interventions studies [20, 21, 53]. The D-galactose-induced accelerated aging rats demonstrated cardiac dysfunction associated with impaired mitochondrial function and autophagy and increased oxidative stress, inflammation, and apoptosis [21], and telomere shortening is considered a hallmark of cardiomyopathies [22]. In the present study, D-galactose-induced accelerated aging mice showed impaired heart function associated with increase in serum AGEs and cardiac telomere length shortening.

We found in D-galactose-induced accelerated aging mice that in addition to the increase of serum AGEs, impaired heart function, and the shortening of myocardial telomere length, the senescence marker proteins p53 and p21 were increased with associated reductions in pAMPK, Sirt6, Sirt1, and NAMPT. All these changes were countered in aging mice treated with oral administration of acacetin in a dose-dependent manner. In H9C2 cardiac cells cultured with D-galactose mitochondrial membrane potential was more depolarized with a decreased mitophagy, which correlates with impaired heart function in aging C57/BL6 mice. Interestingly, in C57/BL6 mice with D-galactose, acacetin reverses the impaired heart function by reducing senescence marker proteins p53 and p21 and enhancing mitophagy (increasing PINK1 and Parkin) in a dose-dependent manner. The mitophagy involvement in acacetin anti-senescence was further confirmed in H9C2 cardiac cells using the autophagy inhibitor 3-methyladenine.

It is generally believed that mitophagy is a selective autophagic removal of mitochondria for clearing away defective mitochondria, which may induce cell damage and death if not well controlled [14]. Therefore, it is important to understand the role of mitophagy in regulating metabolic activity, cell differentiation, apoptosis and other physiological processes of cardiomyocytes and cardiac fibroblasts. PINK/Parkin-mediated mitophagy regulates damage-induced mitochondrial homeostasis [54], and controls mitochondrial quality [55] in various organs/tissues including neuron [56]; decrease in PINK/Parkin correlates with cell senescence. The present study found that PINK1/Parkin and LC3II were remarkably reduced in cardiac tissues and/or cells treated with D-galactose, these reductions were inhibited by acacetin. D-galactose-induced decrease of cardiac mitophagy is associated with inhibition of pAMPK and Sirt6, and acacetin-induced enhancement of mitophagy kinases is related to activation of pAMPK and Sirt6.

**Figure 9:**
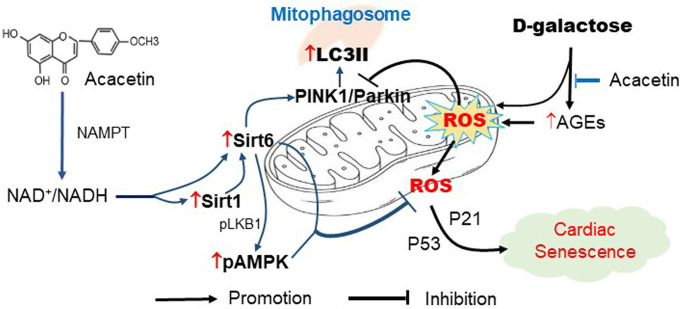
**Schematic signal pathways of D-galactose-induced cardiac senescence and acacetin protection against the cardiac senescence.** D-galactose increases AGEs production and ROS accumulation and decreases mitophagy thereby promoting cardiac senescence. Acacetin, in addition to decreasing AGEs, increases NAD^+^/NADH ratio through NAMPT followed by activation of Sirt1/Sirt6/AMPK thereby preserving mitochondrial function via increasing mitophagy.

Sirt6 is a member of sirtuin family of NAD^+^-dependent deacylases and plays a vital role in chromatin signal transduction and Sirt6-dependent deacetylation is mainly related to the regulation of DNA repair, cellular senescence, lifespan, telomere maintenance and cellular glucose/lipid metabolism [57, 58]. Sirt6 provides protective effect on aging-related cardiovascular disorder by activating autophagy [59, 60]. The present study showed that the protection of acacetin against cardiac senescence was related to Sirt6-mediated protein acetylation inhibition. Silencing Sirt6 abolished acacetin-induced inhibition of protein (e.g. H3K9) acetylation and promotion of the mitophagy kinases PINK1, Parkin, and LC3II. Therefore, Sirt6 is a crucial player in acacetin regulation of mitophagy and inhibition of cardiac senescence induced by D-galactose. Further analysis revealed that Sirt6 activity is closely correlated to activation of NAD^+^/NADH and Sirt1 through NAMPT stimulation (Figure 9).

A number of natural compounds including quercetin, apigenin, resveratrol, naringenin, curcumin, epigallocatechin-3-O-gallate, etc. have been employed experimentally for treating aging-related disorders [61–65]. Experimental studies proved that these compounds were effective in improving structural pathological remodeling, e.g. cardiac and vascular fibrosis, hypertrophy, stiffness, left ventricular dysfunction, and cardiovascular cell death by modifying histone acetylation [62], activating Sirt1/pAMPK [64] or regulating microRNAs [66]. Quercetin at 20 μM improves renal tubular epithelial cell senescence induced by angiotensin II and slowed down renal fibrosis progression by activating Sirt1/PINK1/Parkin pathway [67]. Resveratrol at 25–100 μM attenuates mitochondrial elongation of senescent cardiomyocytes induced by D-galactose via activating Drp1/Parkin/PINK1 mitophagy signals [68]. The present study showed that the concentration (or dosage) range of acacetin for protection against D-galactose-induced cardiac senescence was 1 to 3 μM in cultured H9C2 cardiac cells and 20 to 50 mg/kg/day (orally) in C57/BL6 mice by enhancing mitophagy through Sirt1-mediated activation of Sirt6/AMPK signals. The concentration range of acacetin is clearly lower than quercetin [67] or resveratrol [68], which suggests that acacetin can be a new member of mitophagy enhancers with distinct mechanism and strong pharmacological efficacy.

The present study investigated whether the natural flavone acacetin can protect against cardiac senescence in aging mice induced by D-galactose, in which serum AGEs level was determined, while another D-galactose metabolite galactitol was not measured, this is one limitation of the study. In addition, the evidence that Sirt1-mediated activation of Sirt6 is involved in the protection of acacetin against D-galactose-induced cardiac senescence was determined by silencing Sirt6 and/or Sirt1 in H9C2 cardiac cells, but not in *in vivo* Sirt1 and/or Sirt6 KO animals, which is another limitation of the study. However, this wouldn’t affect the conclusion that protection of acacetin against cardiac senescence induced by D-galactose is mediated by activating the Sirt1/Sirt6/AMPK signal pathway.

Collectively, the present study demonstrates the novel pharmacological effect that the natural flavone acacetin in addition to decreasing AGEs production, protects against cardiac senescence induced by D-galactose with strong efficacy by activating Sirt1 followed by increasing Sirt6/AMPK signals, and thereby enhancing mitophagy function (Figure 9). These results suggest that acacetin may be a drug candidate for treating cardiovascular complications related to aging.

## MATERIALS AND METHODS

### Reagents and antibodies

Acacetin (5,7-dihydroxy-4’-methoxyflavone) used in the present study was synthesized in the laboratory as described previously [40]. Dulbecco’s modified Eagle’s medium (DMEM), Lipofectamine RNAiMAX Reagent and fetal bovine serum (FBS) were purchased from Thermo Fisher Scientific (Waltham, MA, USA), and senescence-associated β-galactosidase (SA-β-gal) staining Kit and Fluorescein di-β-D-galactopyranoside (FDG) were obtained from Sigma-Aldrich (St. Louis, MO, USA). Small-interfering RNAs for Sirt6 and Sirt1were from GenePharma (Shanghai, China). The autophagy inhibitor 3-methyladenine (3-MA) was purchased from MedChemExpress (Shanghai, China). Antibodies used in the present study are listed in Supplementary Table 1.

### Animal experiments

Male C57BL/6 mice (8–10 week-old) were purchased from Wu Experimental Animal Trading Co, Ltd (Fuzhou, China) and maintained under regular light/dark cycle and free access to water and chow diet. The experimental protocol was approved by Animal Use Ethics Committee for Teaching and Research of Xiamen University following the Guidelines on the Use and Care of Laboratory Animals for Biomedical Research published by National Institutes of Health (No. 85–23, revised 1996).

The aging model [20, 21] was induced in C57BL/6 mice by subcutaneous injection of D-galactose (150 mg/kg/day, Sigma Aldrich) or saline (control) for 10 weeks. For drug treatment groups, the D-galactose animals received 10, 20, and 50 mg/kg acacetin daily by intragastric administration, while equivolume vehicle was used for control animals and D-galactose model animals. Echocardiography was performed at end of the experiment, and the animal hearts were dissected, frozen and stored at −80ºC for molecular analysis.

### Echocardiography

Heart function was determined using a Vevo 2100 echocardiograph (VisualSonics Inc., Toronto, Ontario, Canada) in mice anesthetized with 1.5–2.0% isoflurane. Cardiac function and structure were measured with M-mode echocardiography as described previously [19].

### Determination of serum advanced glycation end products (AGEs)

The serum AGEs were determined in each group by enzyme-linked immunosorbent assay using commercial kits (No. CSB-E09414m, Huamei Biotechnology Co. Ltd., Wuhan, Hubei, China) following manufacturer's instructions. In brief, standard or sample was added to each well and incubated 2 hours at 37°C. Then the liquid was removed without washing, Biotin-antibody (1×) was added to each well. After washing for 3 times, avidin horseradish peroxidase (1×) was added to each well for 1 h at 37°C. Then 3,3′,5,5′-tetramethylbenzidine substrate was added for 30 min at 37°C. Finally, stop solution was added to terminate reaction. The plate was read at 450 nm within 5 min, and AGEs level was then calculated.

### Heart section hematoxylin-eosin staining

After reperfusion with normal saline, the isolated mouse heart was weighed, embedded in the optimal cutting temperature compound medium (Sakura Finetek, Japan), then frozen in liquid nitrogen for a few seconds and stored in -80° refrigerator. The heart was sectioned into 6-μm thick slices with a freezing microtome (CM1950, Leica, China). The heart slices were fixed in 4% paraformaldehyde at room temperature for 10 min, washed with running water for 2 min, and stained with hematoxylin-eosin (HE) staining kit (Solarbio, Beijing, China) following the manufacturer’s instructions. The section images were taken with TissueFAXS Plus S (Tissue Gnostics, Austria).

### Cell culture

H9C2 rat cardiomyocyte cell line (ATCC, Manassas, VA, USA) were cultured in Dulbecco’s modified Eagle’s medium (DMEM) with 10% FBS and 1% v/v penicillin/streptomycin at 37°C in 5% CO_2_. When grew to ~80% confluence, the cells were exposed to D-galactose (20 mg/mL) or D-galactose plus acacetin (0.3, 1 or 3 μM) or equivolume vehicle for 72 h. When the siRNA molecules were transfected, cells were treated with Lipofectamine RNAiMAX reagent plus siRNA molecules for 8 h, then exposed to D-galactose in the absence or presence of acacetin for 48–72 h. In the experiments with the NAMPT inhibitor GNE-617 (50 nM) [32] H9C2 cardiac cells were incubated with or without this inhibitor in the absence or presence of 3 μM acacetin for 24 h to inhibit NAD^+^ production.

### Western blot analysis

Western blot analysis was employed to determine specific protein expression with the procedure as described previously [17, 18]. Briefly, total protein was extracted from mouse myocardial tissues, H9C2 cardiac cells or H9C2 cell mitochondria using RIPA lysis buffer with protease inhibitor cocktail (Beyotime, Shanghai, China). Protein concentration was determined using a Bicinchoninic Acid Protein Assay Kit (Solarbio, Beijing, China). The proteins samples were separated through SDS-PAGE and transferred to a polyvinylidene fluoride membrane (Bio-Rad, Hercules, CA, USA). Afterwards, the membranes were blocked with 5% non-fat milk at room temperature for 1 h and incubated with specific primary antibodies as described in Supplementary Table 1 at 4ºC overnight. Membranes were washed three times (10 min each wash) with TBST. The membranes were then incubated with secondary antibodies. After the last wash, the relative expression levels of the proteins were detected using an Enhanced Chemiluminescent detection system. All western blots were repeated at least four experiments, and the signal intensity of the immunoreactive bands was quantified using Image J software (NIH, Bethesda, MD, USA).

### SA-β-gal staining

To estimate cell senescence, senescence-associated β-galactosidase (SA-β-gal) staining was performed following the manufacturer’s instructions. Briefly, H9C2 cardiac cells were seeded in a 6-well plate and grew to ~80% confluence. The cells were treated with D-galactose (20 mg/mL), D-galactose plus acacetin (0.3, 1 or 3 μM) or equivolume vehicle for 72 h. The cells were washed with phosphate buffered saline (PBS) and fixed with fixative solution for 15 min at room temperature. After 3 washes with PBS, the cells were incubated with β-galactosidase staining solution overnight in a dry incubator. Then the cells were observed and images were taken with a light microscope. The percentage of positive cells was calculated by counting the blue-stained cells and total cells (as a standard) in five randomized fields.

### Flow cytometry analysis

Flow cytometry analysis was employed with a flow cytometer (Beckman Coulter, USA) to determine mitochondrial membrane potential and β-galactosidase activity level as described previously [69]. To measure the mitochondrial membrane potential, a JC-1 Mitochondrial Membrane Potential Assay Kit (Solarbio, Beijing, China) was used following the manufacturer’s instructions. Briefly, H9C2 cardiac cells were seeded in a 6-well plate and grew to ~80% confluence. The cells were treated with D-galactose (20 mg/mL), D-galactose plus acacetin (0.3, 1 or 3 μM) or equivolume vehicle for 72 h, and then were incubated with DMEM containing JC-1 (2 μM), a membrane permeable dye for probing mitochondrial membrane potential (Solarbio, Beijing, China), at 37°C for 20 min. Green fluorescence reflected the monomeric form of JC-1, and red fluorescence reflected the aggregate form. Mitochondrial membrane potential was then analyzed with a flow cytometer.

For β-galactosidase activity determination, H9C2 cardiac cells were cultured in the absence and presence of acacetin. After D-galactose exposure, the cells were incubated with 33 μM fluorescein di-β-D-galactopyranoside (FDG) (Sigma-Aldrich) at 37°C for 30 min and intracellular β-galactosidase activity was determined by a flow cytometer.

### Quantitative PCR measurement of telomere length

Telomere length was determined in the cardiac tissue following the procedure as described previously [25]. Briefly DNA was extracted from cardiac tissues using the MiniBEST Universal Genomic DNA Extraction Kit (Takara, Dalian, China), and the telomere length was analyzed using quantitative PCR (qPCR). The sequences (5′−3′) of primers were: Forward−GGTTTTTGAGGGTGAGGGTGAGGGTGAGGGTGAGGGT, Reverse− TCCCGACTATCCCTATCCCTATCCCTATCCCTATCCCTA for telomere; Forward−CACACTCCATCATCAATGGGTACAA, Reverse− CAGTAAGTGGGAAGGTGTACTCA for 36B4. Thermocycling parameters were 95°C for 10 min followed by 40 cycles of 95°C for 15s and 54°C for 60s. All samples were analyzed in triplicate using ABI 7900HT thermal cycler (Applied Biosystems). The relative telomere length was normalized to the internal control 36B4 rRNA.

### Mitochondrial protein extraction

Mitochondria was isolated from H9C2 cardiac cells using a Mitochondria Extraction Kit (Solarbio, Beijing, China) following the manufacturer’s instructions. Briefly, H9C2 cardiac cells were cultured with D-galactose (20 mg/mL) or D-galactose plus acacetin (0.3, 1 or 3 μM) or equivolume vehicle for 72 h. Then, the cells were sonicated and centrifuged to separate cytoplasmic and mitochondrial fractions. The mitochondrial fraction was collected for extracting mitochondrial protein using a RIPA lysis buffer.

### NAD^+^/NADH determination

H9C2 cardiac cells were cultured with or without the NAMPT inhibitor GNE-617 (50 nM) [32] in the absence or presence of 3 μM acacetin for 24 h. Then the cells were harvested and washed with PBS and centrifuged 4 × 1000 rpm for 10 min. The oxidized nicotinamide adenine dinucleotide NAD^+^ and NADH (a reduced form of nicotinamide adenine dinucleotide) levels were quantified using an EnzyChrom™ NAD^+^/NADH assay kit (Bioassay Systems, Hayward, CA, USA) following the manufacturer’s instructions.

### Statistical analysis

Data were expressed as mean ± SEM and analyzed using GraphPad Prism 5.0 software. Multiple group data were statistically analyzed by one way ANOVA followed by Tukey’s post hoc test. A *P* value <0.05 was considered as statistically significant difference.

## Supplementary Materials

Supplementary Figures

Supplementary Table 1
