# Supplementary material for: Cardiac senescence is alleviated by the natural flavone acacetin via enhancing mitophagy
Source: Aging (Albany NY). 2021 Jun 27;13(12):16381–403. doi: 10.18632/aging.203163 (PMC8266317; doi:10.18632/aging.203163)
Supplement: Supplementary Figures [file aging-13-203163-s001.pdf]

SUPPLEMENTARY FIGURES

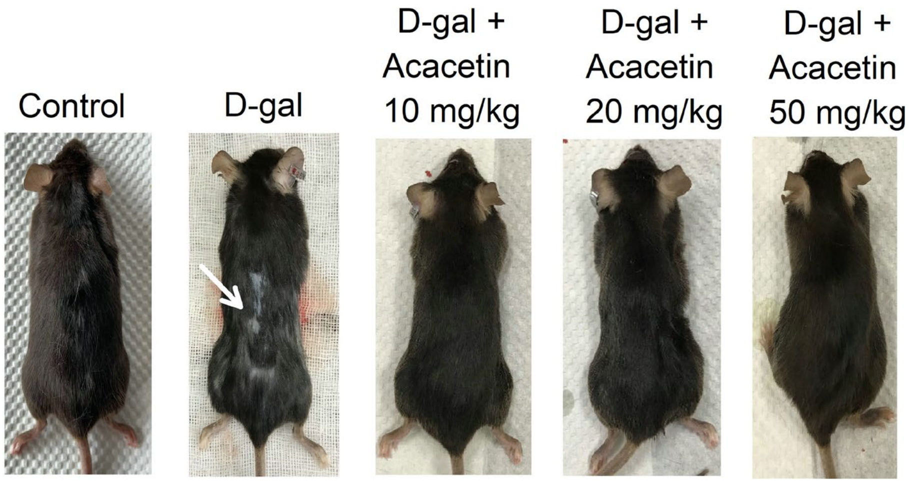

Supplementary Figure 1. Back hair loss was observed in D-galactose-induced accelerated aging mice, but not in animals treated with 10, 20 or 50 mg/kg/day acacetin.

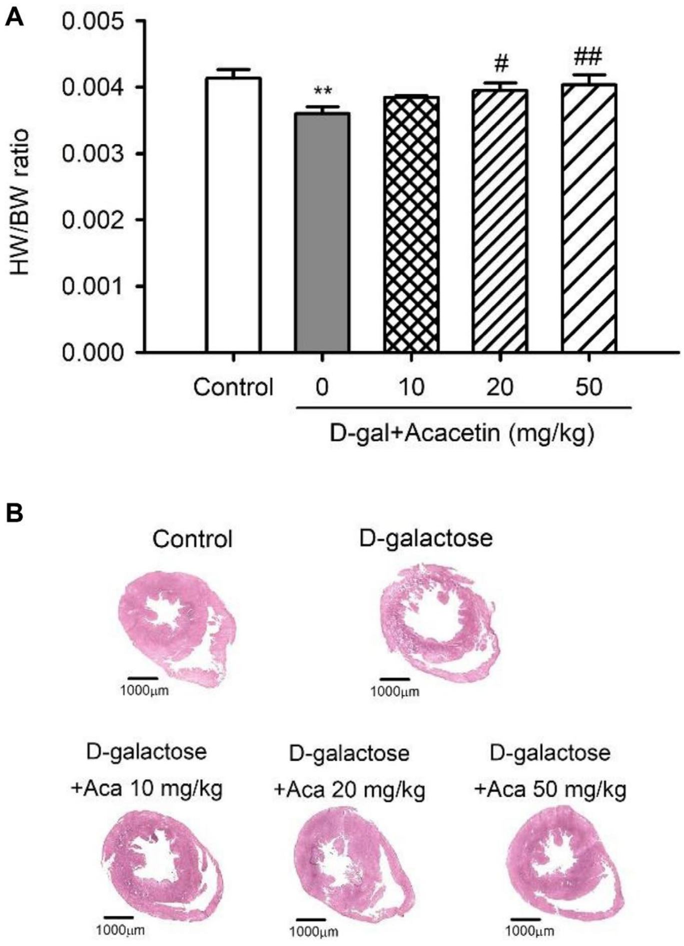

Supplementary Figure 2. Effects of acacetin HW/BW ratio and left ventricular wall in D-galactose-induced accelerated aging mice. (**A**) HW/BW ratio was decreased (n = 8, P < 0.01 vs control) by D-galactose and countered by acacetin in a dose-dependent manner (n = 7–8, $^{\#}P$ < 0.05, $^{\#\#}P$ < 0.01 vs. D-galactose alone). (**B**) Heart sections from control animal, animal with D-galactose, and animals with D-galactose and acacetin (Aca 10, 20, 50 mg/kg/day). HW, heart weight; BW, body weight.

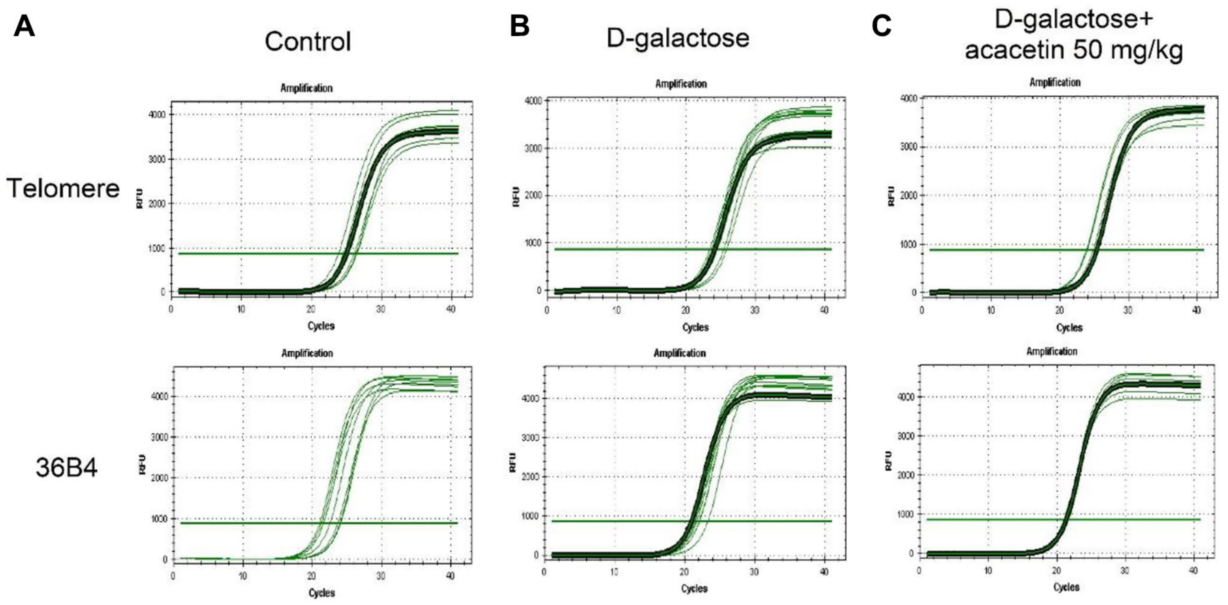

Supplementary Figure 3. Original representative qPCR curves of telomere and 36B4, which was used for calculating relative telomere length, determined in ventricular tissues from a control animal, an animal with D-galactose, and an animal with D-galactose and acacetin (50 mg/kg).

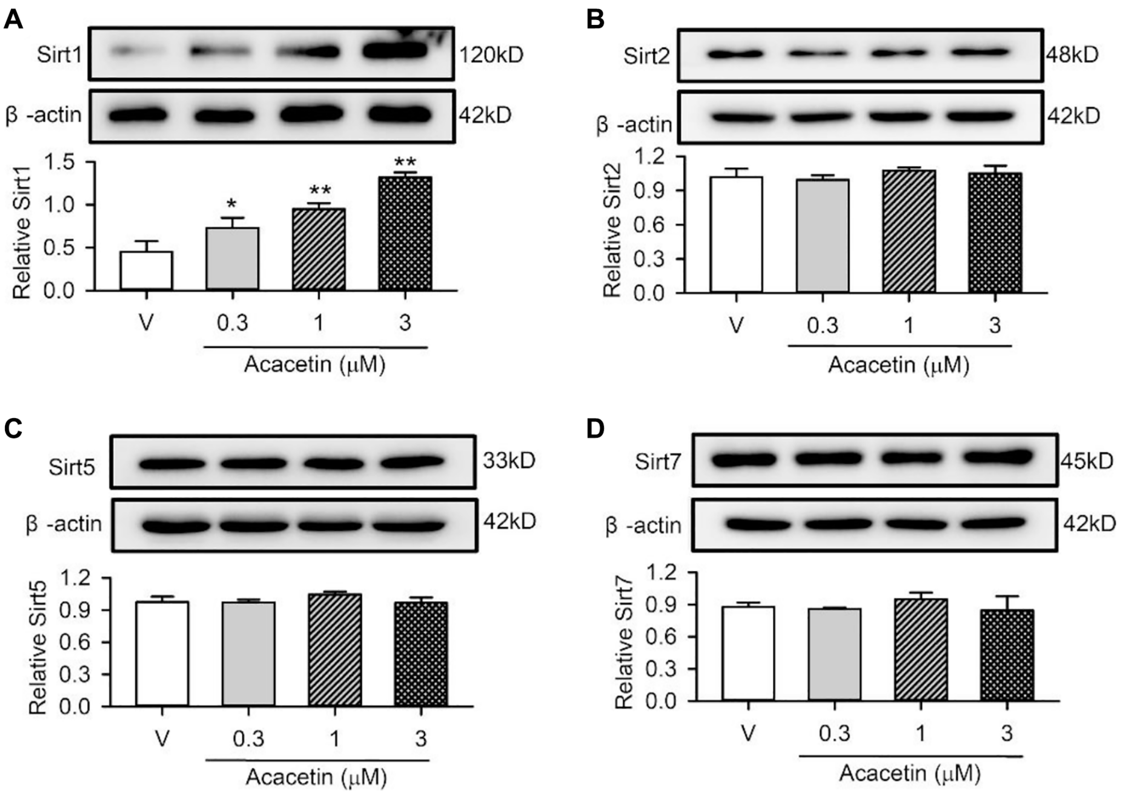

Supplementary Figure 4. Effects of acacetin on expressions of Sirtiuns in H9C2 cardiac cells. (**A**) Sirt1 expression in the absence (vehicle) and presence of 0.3, 1, and 3 μM acacetin (n = 5, $^{*}P < 0.05$, $^{**}P < 0.01$ vs. control). (**B**) Sirt2 expression in the absence (vehicle) and presence of 0.3, 1, and 3 μM acacetin (n = 4, P = NS). (**C**) Sirt5 expression in the absence (vehicle) and presence of 0.3, 1, and 3 μM acacetin (n = 4, P = NS). (**D**) Sirt7 expression in the absence (vehicle) and presence of 0.3, 1, and 3 μM acacetin (n = 4, P = NS).

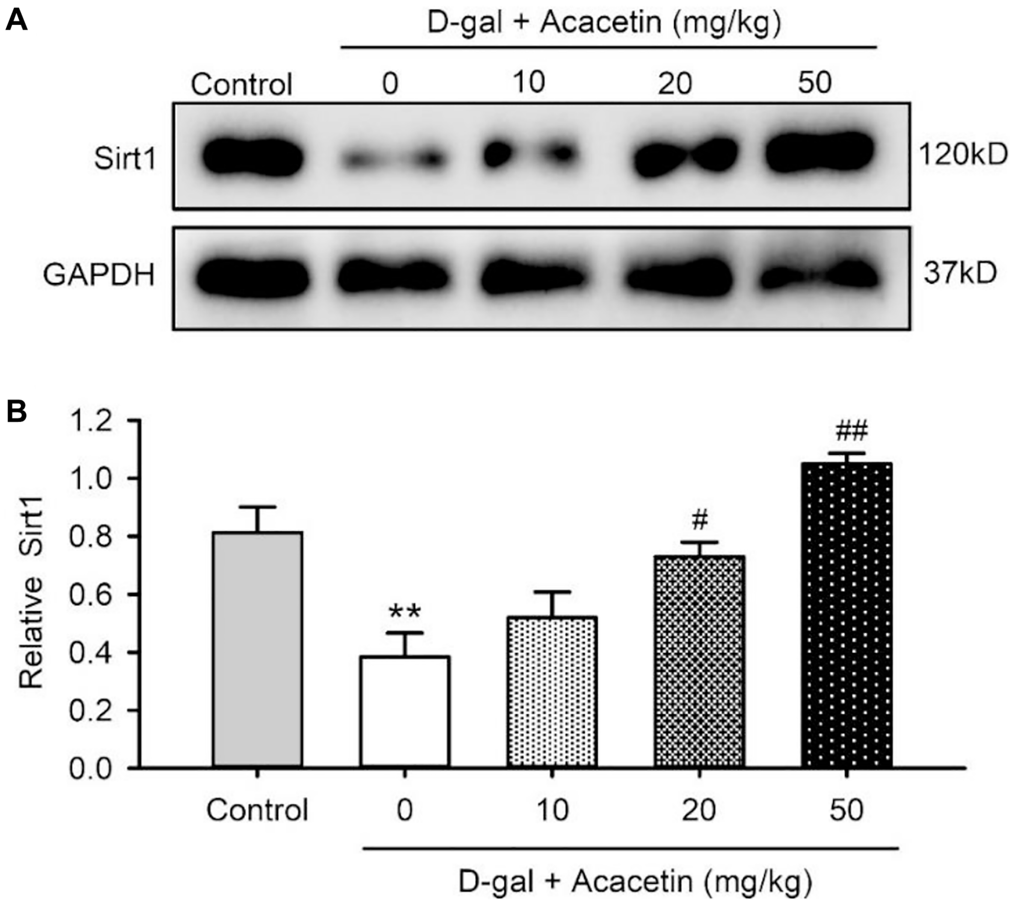

Supplementary Figure 5. Cardiac Sirt1 expression in D-galactose-induced accelerated aging mice without and with acacetin treatment. (**A**). Representative western blots of Sirt1 in cardiac tissues from control animals, animals with D-galactose and animals with D-galactose and acacetin (10 mg, 20 mg, or 50 mg/kg/day). (**B**) Relative Sirt1 levels in cardiac tissues from control animals, animals with D-galactose and animals with D-galactose and acacetin (10 mg, 20 mg, or 50 mg/kg) (n = 5, $^{**}P < 0.01$ vs. control, $^{\#}P < 0.05$, $^{\#\#}P < 0.01$ vs. D-galactose alone).

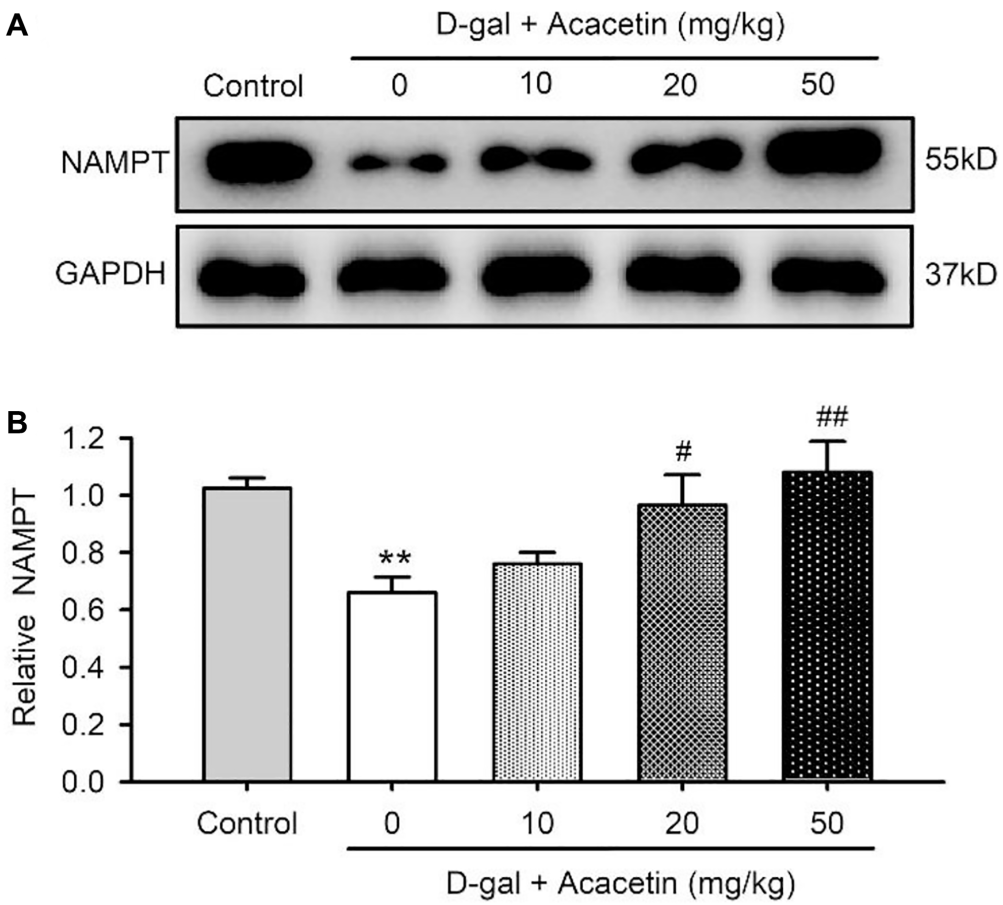

Supplementary Figure 6. Cardiac NAMPT expression from D-galactose-induced accelerated aging mice and acacetin treatment. (**A**) Representative western blots of NAMPT in cardiac tissues from control animals, animals with D-galactose and animals with D-galactose and acacetin (10 mg, 20 mg, or 50 mg/kg/day). (**B**) Relative NAMPT levels in cardiac tissues from control animals, animals with D-galactose and animals with D-galactose and acacetin (10 mg, 20 mg, or 50 mg/kg) (n = 5, $^{**}P < 0.01$ vs. control, $^{\#}P < 0.05$, $^{\#\#}P < 0.01$ vs. D-galactose alone).
