# Supplementary material for: Cardiac senescence is alleviated by the natural flavone acacetin via enhancing mitophagy
Source: Aging (Albany NY). 2021 Jun 27;13(12):16381–403. doi: 10.18632/aging.203163 (PMC8266317; doi:10.18632/aging.203163)
Supplement: Supplementary Table 1 [file aging-13-203163-s002.pdf]

SUPPLEMENTARY TABLE

Supplementary Table 1. Antibody information.

Antibody	Supplier	Cat. number	Dilution
Anti-p53	Cell Signaling Technology (Danvers, MA, USA)	2524T	1:1000
Anti-p21	Proteintech (Rosemont, IL, USA)	28248-1-AP	1:1000
Anti-LC3	Proteintech	18725-1-AP	1:500
Anti-VDAC1	Abcam (Cambridge, MA, USA)	ab154856	1:1000
Anti-Parkin	Cell Signaling Technology	4211T	1:1000
Anti-PINK1	Novus Biologicals (Centennial, CO, USA)	BC100-494	1:1000
Anti-β-actin	Santa Cruz (Dallas, TX, USA)	sc-8432	1:1000
Anti-tAMPK	Santa Cruz	sc-25792	1:1000
Anti-pAMPK	Santa Cruz	sc-33524	1:1000
Anti-NAMPT	Abcam	ab236874	1:1000
Anti-pLKB1	Santa Cruz	sc-271924	1:1000
Anti-tLKB1	Santa Cruz	sc-32245	1:1000
Anti-Sirt1	Abcam	ab189494	1:1000
Anti-Sirt2	Abcam	ab211033	1:2000
Anti-Sirt5	Abcam	ab259967	1:1000
Anti-Sirt6	Abcam	ab191385	1:1000
Anti-Sirt7	Abcam	ab259968	1:1000
Anti-Histone H3	Cell Signaling Technology	4499T	1:2000
Anti-Histone H3 (acetyl K9)	Abcam	ab32129	1:1000
